# Editorial: Brain Organoids: Modeling in Neuroscience

**DOI:** 10.3389/fncel.2020.602946

**Published:** 2020-10-21

**Authors:** Matteo Bordoni, Alysson R. Muotri, Cristina Cereda

**Affiliations:** ^1^Dipartimento di Scienze Farmacologiche e Biomolecolari, Centro di Eccellenza sulle Malattie Neurodegenerative, Università degli Studi di Milano, Milano, Italy; ^2^Department of Pediatrics, University of California, San Diego, San Diego, CA, United States; ^3^Department of Cellular and Molecular Medicine, University of California, San Diego, San Diego, CA, United States; ^4^Genomic and Post-Genomic Center, IRCCS: Istituto di Ricovero e cura a Carattere Scientifico Mondino Foundation, Pavia, Italy

**Keywords:** organoids, editorial, neurological disorder, neurodegenenerative diseases, brain development

The Research Topic “Brain Organoids: Modeling in Neuroscience” collects both original articles and review that highlight the emergency of novel three dimensional (3D) models, a.k.a. brain organoids, for the study of neurological disorders (NDs). Brain organoids are usually generated from pluripotent stem cells (PSCs) and are defined as multi-cellular, 3D, self-assembled, miniaturized structures that mimic the developing human embryonic and fetal brain (Muotri, 2019). Human brain organoids have recently contributed and represent a promising tool to the understanding of several neurological conditions (Chen et al., 2019).

All the articles reported in the Research Topic are a perfect example to understand the potential of brain organoids for new types of analysis.

The main field in which brain organoids were used is the study of neurodevelopment and the disorders connected to it. For example, brain organoids had a pivotal role in studying congenital and acquired microcephaly that occurs during neurodevelopment (Lancaster et al., 2013; Cugola et al., 2016). All the microcephaly studies conducted on brain organoids were reported in detail in the review article of Gabriel et al..

Original data on neurodevelopmental disorders were published by Benson et al., which exploit iPSC-derived brain organoids to demonstrate the common developmental malformation and transcriptional dysregulations that occur on schizophrenic patients with diverse genetic backgrounds. In particular, thanks to the 3D conformation of brain organoids, they evaluated the organoid region that suffers from a disruption in schizophrenic patients, suggesting the fidelity of organoids to model such disease.

Another neurological disability that occurs in early life is the one caused by the prenatal hypoxic injury. In this case, the disorder is not due to genetic factors. Still, it is produced as effects of hypoxia caused by placental insufficiency, umbilical cord occlusion, premature birth, or obstetric complications (Schump, 2018). However, not all the results of hypoxia are currently known, and this is due to the lack of models to study human corticogenesis. With their works, Daviaud et al. reported the data obtained from their implemented model of brain organoids. They concluded that, in the future, their model could be a starting point for testing new therapeutic approaches to both protect and regenerate the most hit cell population during the neurodevelopmental stage.

Brain organoids are also useful to test the fetal cerebral toxicity of small molecules and other chemicals such as in the article of Zhong et al.. In their work, the authors took advantage of the high reliability of organoids to reproduce the human brain fetal development, in contrast to those conducted in rodents, usually more expensive, time-consuming, and do not always represent the real human pathophysiology.

Brain organoids can also be used in the field of neurodegenerative diseases, such as Alzheimer's disease, Parkinson's disease, Amyotrophic Lateral Sclerosis, and Fronto-Temporal Dementia. For example, Simmnacher et al. widely reviewed in their work the limitations that actual models have for the investigation of the pathogenesis of Parkinson's disease. After the review, the authors presented all the work conducted on different types of organoids, highlighting the importance of cell-cell interactions of a 3D model. The authors also showed the extent to optimize actual protocols to obtain the most realistic 3D model of the pathology.

The research article conducted by Nassor et al. shows another issue connected to organoids generation. Indeed, they developed an episomal method to express a plasmid for the mid and long time because standard methods are not suitable for long-term cultures such as organoids. In particular, they used episomal plasmid to generate an isogenic control for the iPSCs-derived organoid culture of Fronto-Temporal Dementia. Interestingly, the approach presented by this work can be successfully applied not only to other neurodegenerative diseases but also to all models based on iPSCs.

One of the most exciting features of brain organoids is their adaptability. Indeed, brain organoids can reproduce different regions of the brain, such as the neocortex (Heide et al., 2018) and midbrain (Smits and Schwamborn, 2020). Moreover, the protocol of brain organoids can be further optimized to obtain unique features (Qian et al., 2019). Chen et al. reviewed the so-called “assembloid,” in which brain organoids are pre-patterned into specific brain regions and fused to model and recapitulate more complex biological processes of human brain development and related diseases. As explained in the review, such an approach was applied to model many processes, i.e., interneuron migration, neuronal projections, tumor invasion, oligodendrogenesis, forebrain axis establishment, and brain vascularization.

Brain organoids can also be engineered at the genomic level. Fischer et al. reviewed this aspect. In their review, the authors explored both stable and transient genetic modification in all the different stages of brain organoids generation, starting from single-cell techniques to fully develop brain organoids. The authors provided an example of adeno-associated virus delivery and electroporation for the transient transfection, and lentivirus delivery, transposon-like systems, and CRISPR/Cas9 for stable transfection.

In conclusion, the Research “Topic Brain Organoids: Modeling in Neuroscience” provides an intriguing overview of the different approaches that can be applied to human brain organoids, suggesting new techniques for the investigation of NDs. We hope that the reader will find in this Research Topic a useful reference for state of the art in the fast-growing field of brain organoids and their use on the study of brain development and NDs.

## Author Contributions

MB wrote the editorial. AM revised the editorial. CC wrote, supervised, and revised the editorial. All authors contributed to the article and approved the submitted version.

## Conflict of Interest

The authors declare that the research was conducted in the absence of any commercial or financial relationships that could be construed as a potential conflict of interest.
